# Navigating *Amaryllidaceae* alkaloids: bridging gaps and charting biosynthetic territories

**DOI:** 10.1093/jxb/erae187

**Published:** 2024-04-23

**Authors:** Nuwan Sameera Liyanage, Fatima Awwad, Karen Cristine Gonçalves dos Santos, Thilina U Jayawardena, Natacha Mérindol, Isabel Desgagné-Penix

**Affiliations:** Department of Chemistry, Biochemistry and Physics, Université du Québec à Trois-Rivières, Trois-Rivières, QC, Canada; Department of Chemistry, Biochemistry and Physics, Université du Québec à Trois-Rivières, Trois-Rivières, QC, Canada; Department of Chemistry, Biochemistry and Physics, Université du Québec à Trois-Rivières, Trois-Rivières, QC, Canada; Department of Chemistry, Biochemistry and Physics, Université du Québec à Trois-Rivières, Trois-Rivières, QC, Canada; Department of Chemistry, Biochemistry and Physics, Université du Québec à Trois-Rivières, Trois-Rivières, QC, Canada; Department of Chemistry, Biochemistry and Physics, Université du Québec à Trois-Rivières, Trois-Rivières, QC, Canada; Plant Biology Research Group, Trois-Rivières, Québec, Canada; La Trobe University, Australia

**Keywords:** *Amaryllidoideae*, *Amaryllidaceae* alkaloids, biosynthesis, galanthamine, *in vitro* culture, isoquinoline alkaloids, multi-omic database, norbelladine, omics, specialized metabolites

## Abstract

*Amaryllidaceae* alkaloid (AA) biosynthesis has garnered significant attention in recent years, particularly with the commercialization of galanthamine as a treatment for the symptoms of Alzheimer’s disease. A significant amount of research work over the last eight decades has focused on the understanding of AA biosynthesis, starting from early radiolabelling studies to recent multi-omics analysis with modern biotechnological advancements. Those studies enabled the identification of hundreds of metabolites, the characterization of biochemical pathways, and an understanding of the environmental stimuli and of the molecular regulation of these pharmaceutically and agriculturally important metabolites. Despite numerous studies, there remain significant gaps in understanding the biosynthesis of AAs in *Amaryllidaceae* plants. As such, further research is needed to fully elucidate the metabolic pathways and facilitate their production. This review aims to provide a comprehensive summary of the current state of knowledge on AA biosynthesis, from elicitation of expression of transcription factors in the cell nucleus to alkaloid transport in the apoplast, and to highlight the challenges that need to be overcome for further advancement.

## Teaser

This review explores the landscape of *Amaryllidaceae* alkaloid biosynthesis, tracing its evolution from radiolabelling studies to multi-omics analyses, emphasizing gaps and challenges, and urging further research to unlock their full potential.

## Introduction

Tens of thousands of years of human civilization have depended on nature to grant the cure for illnesses and diseases. *Amaryllidaceae* J. St.-Hil. (*sensu stricto*) is a plant family that has provided beneficial medicinal value worldwide (Jin and Yao, 2019). Various ethnic groups have traditionally used this plant family to treat a range of illnesses, such as mental health issues, cancer, and respiratory and liver problems (Nair and van Staden, 2013). For instance, *Crinum zeylanicum* has been used in Sri Lankan folk medicine to treat rheumatism, snake-bites, and ear-aches (Jayaweera, 1981; Tsuda *et al.*, 1984), while *Zephyranthes fosteri* was used in traditional Aztec medicine to treat ‘fatigue’ and ‘stress’ (Centeno-Betanzos *et al.*, 2021). Chinese folk medicine has long utilized *Lycoris radiata* bulbs to treat skin and laryngeal conditions, while *Amaryllis belladonna* and *Boophone disticha* have been used in the African continent to treat cancer, inflammation, wounds, and infections (Wang *et al.*, 2009; Nair and van Staden, 2013). The *Amaryllidaceae* J. St.-Hil., also known as subfamily *Amaryllidoideae* according to APG III, is a cosmopolitan family of bulbous monocots consisting of ~900 species shared by ~75 genera, that thrive mainly in Africa and South America (Meerow *et al.*, 1999; APG, 2009). Their slow-blooming, exquisite flowers render them popular in horticulture. Their ability to produce a unique group of alkaloids called the *Amaryllidaceae* alkaloids (AAs) may explain the use of these geophytes in traditional medicine systems around the world.

AAs are basic nitrogen-containing specialized metabolites with a benzopyridine heterocyclic group. More than 650 different AAs have been elucidated so far. They derive from the metabolism of phenylalanine and tyrosine (Desgagné-Penix, 2021; Jayawardena *et al.*, 2024). In the plant, they display defence properties to protect against abiotic or biotic stress, such as predators, targeting their nervous system (Berkov *et al.*, 2020; Nair and van Staden, 2020), or to attract pollinators to promote seed dispersion (Berkov *et al.*, 2020). Therapeutically, AAs exhibit various biological activities, including anti-acetylcholinesterase, antiviral, antibacterial, antifungal, anticancer, and cytotoxic activities (Ding *et al.*, 2017; Hotchandani and Desgagne-Penix, 2017; Berkov *et al.*, 2020; Ka *et al.*, 2020). This wide range of potential pharmaceutical applications position AAs as attractive candidates for the development of new drugs. One of the main breakthroughs has been the approval of galanthamine as a treatment of mild symptoms of cognitive impairment and Alzheimer’s disease in at least 29 countries (Olin and Schneider, 2002; Loy and Schneider, 2006). Galanthamine selectively, reversibly, and competitively inhibits acetylcholinesterase, which leads to improved cognitive function (Olin and Schneider, 2002). Lycorine and its derivatives also attracts a lot of attention due to their strong anticancer properties (Roy *et al.*, 2018). Many other AAs, such as cherylline, crinamine, and pancratistatin, are effective against multiple viruses, including herpes simplex virus, Rauscher leukaemia virus, coronaviruses, flaviviruses, human immunodeficiency virus, and hepatitis C virus, as reviewed in Jayawardena *et al.* (2024). Narciclasine, lycorine, and diverse AAs also display antifungal activities through a plethora of mechanisms (Nair and van Staden, 2020). Cripowellin, lycorine, ungeremine, and multiple AAs exhibit antibacterial activity, and are studied pharmacologically to overcome antibiotic resistance (Bendaif *et al.*, 2018; Chen *et al.*, 2018; Kianfe *et al.*, 2020). AAs such as crinamine, 8α-ethoxyprecriwelline, epivittatine, lycorine, and derivatives are evaluated for their anti-inflammatory activity specific to cyclooxygenese-1 and -2 (Elgorashi *et al.*, 2003; He *et al.*, 2015). Their multifaceted activities highlight the relevance of these plants’ metabolites in drug discovery for improvement of human health.

The yield of an AA of interest is limited and variable in plants grown in the wild, in part due to the diversity of plant metabolic routes and to environmental stresses. In the wild, *Amaryllidaceae* grow in specific regions, sometimes under singular conditions, and some are classified as endangered species, such as *Eucrosia stricklandii*, a rare *Amaryllidaceae* from Ecuador, while several *Narcissus* species have already become extinct (Colque *et al.*, 2002; Santos-Gally *et al.*, 2015). In a nutshell, wild plants are not a suitable sustainable source of medicinal compounds. Organic synthesis is a challenging, less profitable, and not a sustainable alternative because of the complexity of the structure of AAs (Kohelová *et al.*, 2021). Usually, they are extracted directly from *Amaryllidaceae* harvested from the field or greenhouse, or micropropagated, but the AA yield is low. Much effort is concentrated on developing *in vitro* systems with profitable production of AAs, and in uncovering biosynthetic pathways to acquire the knowledge to carry out metabolic engineering (Koirala *et al.*, 2022), but much remains to be discovered with regards to their biosynthesis, and transport in their natural host.

A growing number of research papers have been exploring different aspects of AA biosynthesis, such as enzyme discovery, substrate selectivity, and pathway hypothesis. In this review, we will summarize the established biosynthetic steps, examine both *in vivo* and *in vitro* plant studies that helped unravel enzymatic reactions or their regulation, and outline the available multi-omics data. Additionally, we will discuss the latest insights into the pathway characteristics *in planta* and explore modern techniques and tools that can expedite pathway assembly.

## Structural diversity of *Amaryllidaceae* alkaloids

Ever since lycorine was isolated from *Narcissus pseudonarcissus*, 150 years ago, scientists have identified and determined the structures of hundreds of AAs (Gerrard, 1877; Ding *et al.*, 2017). Each year, several new alkaloids from *Amaryllidaceae* species are added to the list, making the puzzle of their biosynthetic route increasingly complex. For instance, in 2023 and 2024, Chaichompoo and colleagues reported 18 new AAs from the bulbs of *Crinum latifolium* and *Crinum×amabile* (Chaichompoo *et al.*, 2023, 2024). Experts in the field of AAs have suggested various classification systems for their structure. One of the earliest classifications was introduced by Wildman, based on the presence of different types of nucleus, such as pyrrolo [*de*] phenanthridine, dibenzofuran, or [2] benzopyrano [3,4*g*] indole (Wildman, 1960). Later, Ghosal *et al.* presented a 12-ring system, which is still considered as the standard for numbering ring carbons (Ghosal *et al.*, 1985). Several other ring-type classification systems were proposed based on chemical structure analysis or AA biosynthetic origin, including 42 by Berkov *et al.* (2020), nine by Bastida *et al.* (2006), 18 by Jin (2009), and nine by Desgagné-Penix (2021). These classification systems evolve through years with the periodical discovery of novel alkaloids. Hence, there is currently no universal classification system for AAs.

### *Amaryllidaceae* alkaloid biosynthesis

Despite divergences in the classification systems of AA complex structures, the general early steps of their biosynthesis, and the precursors involved, namely tyramine and 3,4-dihydroxybenzaldehyde (3,4-DHBA) coming from the phenylpropanoid pathway, are largely accepted (Kilgore and Kutchan, 2016; Desgagné-Penix, 2021). A tyrosine decarboxylase (TYDC) catalyses decarboxylation of tyrosine into tyramine, as studied in *Narcissus* aff. *pseudonarcissus*, *Lycoris radiata*, and *L*. *aurea* (Kilgore, 2015; Wang *et al.*, 2019; Hu *et al.*, 2021); while a phenylalanine ammonia-lyase (PAL) explored in *L*. *radiata*, and a cinnamic acid 4-hydroxylase (C4H) uncovered in *L*. *radiata* and *L*. *aurea* catalyse important steps of the phenylpropanoid pathway (W. Li *et al.*, 2018; Y. Li *et al.*, 2018). There remain gaps in knowledge of the precursor pathway such as the synthesis of 3,4-DHBA which still awaits being uncovered. Nevertheless, these steps are beyond the scope of this review which focuses on the biosynthesis of AAs, starting from the condensation step.

### Early and current evidence for biosynthesis of *Amaryllidaceae* alkaloids

Analytical techniques such as HPLC, GC, MS, and NMR, and *in situ* metabolite imaging techniques such as matrix-assisted laser desorption/ionization (MALDI) and desorption electrospray ionization- (DESI) coupled MS have allowed the detection and the elucidation of AA structures and their localization *in planta* (Kilgore *et al.*, 2014; (Mehta *et al.*, 2024). In early studies dating back to the 1950s, radioisotope studies contributed to the identification of precursors and intermediates, and to the assembly of AA metabolic pathways (Barton and Cohen, 1957). Radioactive or stable isotope labelling, random mutagenesis with ethyl methanesulfonate (EMS) or γ-radiation, gene silencing, and multi-omics techniques all contributed to identify specific gene and enzyme candidates. Their integration is decisive to metabolite pathway elucidation. For instance, this approach has enabled the assembly of the canonical pathway of vincristine and vinblastine from *Catharanthus roseus*, a well-studied medicinal plant, but it took >30 years (Qu *et al.*, 2019).

In this review, the AA pathway will be divided into three sections: ‘Formation of the initial stable intermediates’; ‘Oxidative phenol coupling for diversification of the metabolites’; and ‘Downstream pathways’.

#### Formation of the initial stable intermediates

**Fig. 1.**
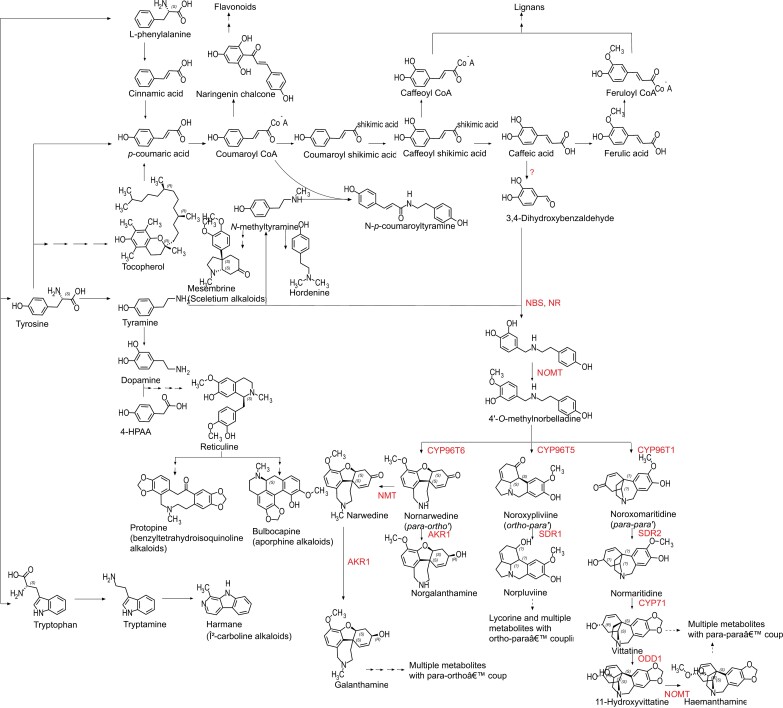
*Amaryllidaceae* metabolic pathways. Enzymatically characterized steps of the *Amaryllidaceae* alkaloid (AA) pathway along with other defence-related specialized metabolites and non-AA representatives recorded in the family starting from the three aromatic amino acids. A single arrow represents a single biochemical step, while multiple arrows represent multiple steps. Identified enzymes in the AA pathway are represented in red. Abbreviations are in the text.

*The formation of norbelladine*. In the 1960s, the early radioactive isotope labelling studies gave the first insight that tyramine, as the amine group, and 3,4-DHBA, as the aldehyde partner, were incorporated into multiple AAs such as lycorine, haemanthamine, and galanthamine (Suhadolnik *et al.*, 1962, 1963; Wildman *et al.*, 1962; Feinstein, 1967). Another radiolabel study showed that the two precursors combined to yield norbelladine, as a scaffold reaction for the biosynthesis of all AAs (Battersby *et al.*, 1961). Hence, to understand AA biosynthesis, the formation of norbelladine is the first key reaction to explore. Biochemically, norbelladine is synthesized through a reduction reaction that follows condensation of tyramine and 3,4-DHBA yielding norcraugsodine, a Schiff base (Fig. 1) (Majhi *et al.*, 2023).

The first enzymatic evidence for the formation of norbelladine was reported by Kilgore *et al.* (2016b). From the transcriptome of *Narcissus* aff. *pseudonarcissus*, they identified and cloned a short chain dehydrogenase (SDR) named noroxomaritidine/norcraugsodine reductase (NR), which is phylogenetically close to a tropinone reductase II from *Datura stramonium* producing tropane alkaloids. Although the major reaction catalysed by that enzyme is related to a downstream pathway step, NR can catalyse the reduction of norcraugsodine to norbelladine, using tyramine and 3,4-DHBA as substrates. Later, Singh *et al.* (2018) identified another candidate for this reaction from the *Narcissus pseudonarcissus* transcriptome. Norbelladine synthase (NBS) is an orthologue to norcoclaurine synthase (NCS), a well-characterized enzyme that catalyses the first condensation reaction in the benzylisoquinoline alkaloid (BIA) pathway via a Pictet–Spengler reaction. NBS from *N*. *pseudonarcissus* rather commits to a Mannich reaction for the condensation of tyramine and 3,4-DHBA (Singh *et al.*, 2018). Orthologous NBS enzymes from *Leucojum aestivum* and *Narcissus papyraceus* were cloned and shown to catalyse the same reaction (Tousignant *et al.*, 2022; Majhi *et al.*, 2023). Interestingly, norbelladine synthesis yield is increased through the interaction between NBS and NR, that catalyse the condensation and the imine reduction, respectively, possibly through preventing the degradation of the unstable norcraugsodine (Fig. 1) (Majhi *et al.*, 2023). None of the studies related to NBS or NR provides any information related to enzyme kinetics. This will be necessary for a better biochemical understanding of the reactions. Nonetheless, NBS and NR may be the gateway to the formation of AA, catalysing the first steps in their biosynthesis. Similar to the formation of norcoclaurine in the BIA pathway or of the 1-phenethylisoquinoline scaffold in phenethylisoquinoline (PIA) alkaloids, the synthesis of norbelladine is the step that establishes the structural features observed in AAs (Beaudoin and Facchini, 2014; Nett *et al.*, 2020).

*The formation of 4'-O-methylnorbelladine*. Norbelladine 4'-*O*-methylation is another key step of the biosynthesis of AAs, because it is required for the subsequent oxidative phenol coupling (Kilgore *et al.*, 2014). 4'-*O*-Methylnorbelladine was established as a central intermediate by radioisotope labelling experiments that aimed to uncover the origin of the methylenedioxy group in haemanthamine (Barton *et al.*, 1962a). Mann *et al.* (1963) also provided the first evidence of a norbelladine-*O*-methyltransferase (N*O*MT) that catalysed this reaction, by identifying an analogous enzyme to a catechol *O*-methyltransferase (C*O*MT) partially purified from *Nerine bowdenii*. This study established 4'-*O*-methylnorbelladine as the intermediate required for the downstream pathway. Thirty-five years later, a study that focused on galanthamine biosynthesis confirmed the catalysis of norbelladine *O*-methylation using crude enzyme extracts from six *Amaryllidaceae* species, with the recurrent observation that 4'-*O*-methylation was favoured over 3'-*O*-methylation (Mann *et al.*, 1963; Eichhorn *et al.*, 1998). The proteins extracted from the leaves of *Clivia miniata* and *Leucojum vernum* demonstrated the best activity (Eichhorn *et al.*, 1998). Sixteen years further on, and the first N*O*MT, classified as a class I *O*-methyltransferase, was cloned and a detailed characterization of the step was performed during the assembly of the *N.* aff. *pseudonarcissus* transcriptome (Fig. 1) (Kilgore *et al.*, 2014). Several orthologues with various substrate specificities and regioselectivities were characterized from *Lycoris radiata*, *L*. *aurea*, and *L*. *longituba* (Sun *et al.*, 2018; Li *et al.*, 2019; Li *et al.*, 2020). The W50M/A53N/Y186K triple variant of *Galanthus elwesii* N*O*MT showed that the inversion of regioselectivity from 1:99 to 94:6 (*para*/*meta*) can be achieved through specific mutations and coordinating Ni^2+^ instead of Mg^2+^ as the metal ion partner (Su *et al.*, 2022).

##### Norbelladine or 4'-*O*-methylnorbelladine as key intermediates

Although early isotope labelling studies established norbelladine as the first intermediate for the downstream pathways, the literature related to N*O*MT promiscuity raises a doubt about the order of the reactions leading to the formation of norbelladine and its methylated form. Indeed, the first N*O*MT candidate studied in 1963 was shown to methylate dopamine, which is a hydroxylated form of tyramine (Mann *et al.*, 1963). N*O*MTs from *L*. *radiata* and *L*. *aurea* catalyse the *O*-methylation of 3,4-DHBA and caffeic acid in addition to norbelladine. Hence, other precursors, such as vanillin and isovanillin (the methylated products of 3,4-DHBA), could condense with tyramine to yield 3'-*O*-methylnorbelladine and 4'-*O*-methylnorbelladine, respectively, suggesting a more complex metabolic route (Sun *et al.*, 2018; Li *et al.*, 2019). Furthermore, *O*-methylation of other norbelladine derivates such as *N*-methylnorbelladine was observed in some of the studies mentioned above (Eichhorn *et al.*, 1998; Kilgore *et al.*, 2014). NR was shown to catalyse the condensation and reduction of isovanillin and tyramine to form 4'-*O*-methylnorbelladine, but a lower yield was observed compared with norbelladine synthesis (Kilgore *et al.*, 2016b). The ability of NBS to catalyse the condensation of methylated precursors, such as vanillin and isovanillin, with tyramine remains to be tested.

#### Oxidative phenol coupling to branch into multiple directions

In plants, phenol coupling is observed in the synthesis of various specialized metabolites including lignans, flavonoids, and alkaloids. It is a key step in the synthesis of AAs. Following this reaction, the basic alkaloid structures undergo further changes to produce a range of distinct alkaloid compounds. Oxidative phenol coupling involves the formation of C–C and C–O bonds primarily catalysed by cytochrome P450 (CYP), with laccases and peroxidases playing a role in some cases. These enzymatic reactions are highly regio- and stereoselective, contributing significantly to the production of specialized metabolites (Hüttel and Müller, 2021). Barton and Cohen (1957) were the first to show evidence of phenol oxidation in the formation of AAs. They proved through radiolabelled studies that 4'-*O*-methylnorbelladine, not *O*,*N*-dimethylnorbelladine or *N*-methylnorbelladine, underwent the phenol coupling reaction to synthesize AAs, such as galanthamine and haemanthamine, in *N*. *pseudonarcissus* (Barton and Kirby, 1962; Barton *et al.*, 1963). Depending on the C–C bond formation, three types of phenol couplings were suggested, namely *para–ortho*ʹ, *ortho–para*ʹ, and *para–para*ʹ (Barton *et al.*, 1962b, 1963; Fuganti and Mazza, 1971; Eichhorn *et al.*, 1998). The formation of these bonds and the details of the biochemical reactions are well described in various literature reviews (Bastida *et al.*, 2006; Jayawardena *et al.*, 2024).

Transcriptome assembly from *N*. aff. *pseudonarcissus* and correlation analysis with N*O*MT led to the first characterization of gene candidates categorized under a novel CYP subfamily, named CYP96T (Kilgore *et al.*, 2016a). Enzymatic characterization of the candidate CYP96T1 showed that this enzyme catalyses *para*–*para*ʹ coupling [(10b*S*,4a*R*)- and (10b*R*,4a*S*)-noroxomaritidine] of 4'-*O*-methylnorbelladine as the major reaction (Fig. 1), leading to AAs such as crinine, montanine, and pretazzetine, and *para–ortho*ʹ phenol coupling (nornarwedine) as a minor reaction, leading to galanthamine (Kilgore *et al.*, 2016a). *In silico* modelling, dynamics, and simulations provided an atomic understanding of the C–N coupling, and C–C bond formation that follows a diradical mechanism in the active site of CYP96T1 (W. Peng *et al.*, 2023).

Recently, Mehta and colleagues conducted an interesting study that expanded our understanding of CYP96T enzymes in AA biosynthesis (Mehta *et al.*, 2024). They used a combined approach of stable isotope labelling and transcriptome analysis, followed by co-expression analysis, to identify potential genes for the phenol coupling of 4'-*O*-methylnorbelladine. Their research suggests that three different CYP96T types are involved in *para–ortho*ʹ, *ortho–para*ʹ, and *para–para*ʹ phenol couplings. They confirm that CYP96T1 catalyses *para–para*ʹ coupling, and propose that CYP96T6 leads to *para–ortho*ʹ phenol coupling, while CYP96T5 catalyses *ortho–para*ʹ coupling (noroxopluviine) leading to lycorine-type AAs (Fig. 1). They also present evidence that these enzymes could be modified to alter their regioselectivity; that is, substituting Leu308 with alanine on the *para–para*ʹ coupling enzyme CYP96T1 yielded the same catalytic capacity as the *para–ortho*ʹ oxidative coupling enzyme CYP96T6. If confirmed, these results will shed light on the divergent regioselectivity of CYP96T, and on the means to achieve AA molecular diversity.

*Paths to galanthamine, haemanthamine, and lycorine*. Intermediate compounds formed by the oxidative phenol couplings undergo further chemical changes, such as hydroxylation, methylation, reduction, oxidation, condensation, and oxygen bridge formation (Kilgore and Kutchan, 2016). Early isotope labelling and organic synthesis have helped build up multiple hypotheses for the synthesis of the intermediate and downstream metabolites, providing the basis to interpret the biosynthetic path of newly discovered compounds (Barton *et al.*, 1962a; Eichhorn *et al.*, 1998; Berkov *et al.*, 2020). An alternative way has been to compare structures and reactions of the AA pathway with specialized metabolic pathways from other plant families, as this provides strong hints on the candidate enzymes. For example, enzyme families such as aldo–keto reductases (AKRs), SDRs, CYP450 monooxygenases such as CYP71, *O*- and *N*-methyltransferases (*O*MT, *N*MT), and 2-oxoglutarate-dependent dioxygenases (ODDs) are known plant enzyme superfamilies which catalyse multiple biochemical reactions diversifying alkaloid structures (Kilgore and Kutchan, 2016).

The first molecular evidence of enzymes involved in the AA downstream pathway came from studies of NR catalysing noroxomaritidine to oxomaritine, and of vittatine 11-hydroxylase catalysing vittatine to 11-hydroxyvittatine (Kilgore, 2015; Kilgore *et al.*, 2016b). Vittatine 11-hydroxylase is an ODD homologous to an enzyme characterized in *Pisum sativum* that produces gibberellin (Kilgore, 2015). Isotope feeding of multiple tissue sections of *Narcissus* cv. ‘Tête-à-Tête’ and correlation analysis of the transcriptome suggested the involvement of multiple enzymes, such as SDR, AKR, *O*MT, *N*MT, CYP71, and ODD, in the synthesis of haemanthamine and galanthamine from 4'-*O*-methylnorbelladine (Fig. 1) (Mehta *et al.*, 2024). The *O*MT proposed to catalyse the *O*-methylation of 11-hydroxyvittatine to yield haemanthamine is an orthologue to N4*O*MT, and the NMT catalysing nornarwedine to narwedine is a γ-tocopherol methyltransferase, homologous to an enzyme involved in colchicine synthesis (Nett *et al.*, 2020; (Mehta *et al.*, 2024). As the information regarding the transcripts and amino acid sequences is not yet available, it is difficult to discuss these enzymes, their mechanism, or their phylogenetic relationships further.

### Future directions for enzyme discovery

On the path to discover novel enzymes, several future directions should be considered, including deepening our knowledge of already characterized steps. Further research should focus on testing and validating experimentally various hypotheses, such as resolving the involvement of multiple precursors in the formation of the first intermediates (Li *et al.*, 2019). Furthermore, it will be important to study substrate specificity and promiscuity of *O*- and *N*-methyltransferases, hydroxylases, and dehydrogenases discovered in the early pathway, in the context of downstream steps.

#### Involvement in defence and in other pathways

The precursor pathway, which consists primarily of the phenylpropanoid pathway and tyramine, is not only responsible for the synthesis of alkaloids in plants, but also contributes to the production of other defence chemicals, such as lignans, flavonoids, and coumarins (Fig. 1) (de Vries *et al.*, 2021). The phenylpropanoid pathway has multiple functions, highlighting the versatility of plant metabolic routes and their importance in protecting plants against herbivores and pathogens, helping them adapt to various environmental conditions (Dong and Lin, 2021). Understanding the synchronization of the production of different classes of specialized metabolites may help in discovering promiscuous enzymes which co-evolved in these multiple branches. For example, *C*. *roseus* 16-hydroxytabersonine-*O*-methyltransferase catalyses the *O*-methylation of both flavonoid and alkaloid synthesis, giving some insights into the evolutionary relationships of multiple pathways related to plant chemical defences (Lemos Cruz *et al.*, 2023).

There could be relationships not only with non-alkaloid pathways, but also between some major AA groups or with other alkaloids. Cherylline and norbelladine, which do not undergo phenol coupling, could have evolved independently from other AA groups (Desgagné-Penix, 2021; Jayawardena *et al.*, 2024). Moreover, there are other alkaloid groups reported in the *Amaryllidaceae* family, such as sceletium, phthalideisoquinoline, benzyltetrahydroisoquinoline, β-carboline, and aporphine alkaloids, also produced by other plant groups, such as *Sceletium*, *Papaveraceae*, and *Fumariaceae* (Fig. 1). The elucidated pathways of these multiple non-AA groups may help identify more candidate enzymes associated with the production of AAs.

Studying evolution of plant pathways would contribute to reinforce our knowledge on AA biosynthesis *in planta*. There are studies on the evolutionary origin of a few alkaloid groups such as BIA in the plant kingdom, yet no studies are available for AAs, with the exception of some studies on alkaloid diversity within the family, or within a genus such as *Narcissus* (Liscombe *et al.*, 2005; Rønsted *et al.*, 2012; Berkov *et al.*, 2014). By investigating across different plant species, we can also gain new insights, improve our understanding of AAs, and provide a broader perspective on alkaloid biosynthesis in plants in general. For instance, as the biosynthesis of norbelladine follows a similar pathway to that of BIAs and PIA, this suggests a possible shared evolutionary history or convergent evolution.

Recently, transient expression approaches such as agroinfiltration and viral-induced gene silencing (VIGS) contributed to the discovery of specialized metabolite biosynthesis pathways, such as colchicine biosynthesis, and to the discovery of a serpentine synthase gene in *C*. *roseus* (Nett *et al.*, 2020; Yamamoto *et al.*, 2021). In AA biosynthesis, agroinfiltration was only used in one study, producing galanthamine and haemanthamine in *Nicotiana benthamiana* (Mehta *et al.*, 2024). Application and establishment of VIGS in *Amaryllidaceae* plants were conducted in *Narcissus tazetta* for silencing *MYB3* in relation to flavonoid biosynthesis, and in *Lycoris chinensis* for silencing *Cloroplastos Alterados 1* (*CLA1*) and *Phytoene Desaturase* (*PDS*) genes. However, the use of VIGS for characterizing AA biosynthesis has not yet been reported (Zhou *et al.*, 2021; Cheng *et al.*, 2023).

#### Directions for further characterizations of enzymes

Enzyme structure is elucidated through techniques such as X-ray crystallography, Raman spectroscopy, or cryo-EM. There is a scarcity of enzyme crystal structures in AA biosynthesis which makes it difficult to understand their molecular mechanisms. Currently, there is only one crystallized structure of an enzyme related to an AA pathway in the Protein Data Bank (PDB), namely NR from *N*. aff. *pseudonarcissus* in complex with NADP+ and tyramine or other substrates (PDB: 5FEU, 5FF9, 5FFF) (Kilgore *et al.*, 2016a). A conference abstract mentions the elucidation of the crystal structure of N4*O*MT from *L. longituba*, but there is no further information as of yet (Hnin *et al.*, 2023). Advanced protein structure prediction tools such as DeepMind Alphafold2 will contribute to overcome the gap of the availability of crystallized structures (Jumper *et al.*, 2021; C.-X. Peng *et al.*, 2023). Although those tools are not a replacement for experimental evidence, examples such as the molecular dynamics of CYP96T1 and prediction of the effects of mutations on the inversion of regioselectivity of N*O*MT were achieved based on the protein structures predicted by Alphafold (Su *et al.*, 2022; W. Peng *et al.*, 2023).

In addition, detailed kinetic studies are required to improve our understanding of the catalytic efficiencies, substrate specificities, and regulatory mechanisms. Only then can enzyme activity be optimized with directed evolution and rational design. Surprisingly, not much effort has been put into improving the activity of enzymes that are responsible for producing alkaloids in *Amaryllidaceae*, with the exception of N*O*MT engineered by Su *et al.* (2022). Such research may lead to the development of biotechnological approaches for increasing the production of specific alkaloids or even the creation of new compounds (Boccia *et al.*, 2022).

## Cellular and tissue organization of the pathway

This section focuses on the molecular regulation and organization of the pathway. Understanding the mechanisms that regulate metabolite biosynthesis at the plant and cell level is crucial for its advancement. Unlike some other well-studied medicinal plants, there is limited literature available for the *Amaryllidaceae* family.

### Organ-specific expression and subcellular localization of NBS, NR, N4*O*MT, and CYP96T

**Fig. 2.**
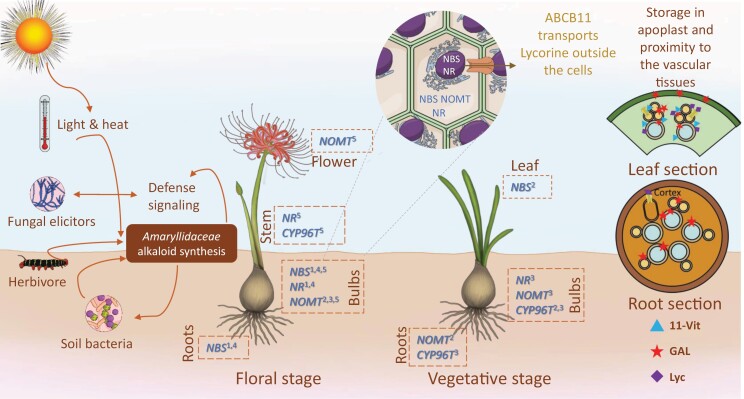
A summary of *Amaryllidaceae* alkaloid (AA) metabolism, representing environmental stimuli initiating the biosynthesis, gene expression, and protein expression of genes associated with the AA pathway, and accumulation of a few AAs at the tissue level. ^1^*Leucojum aestivum*, ^2^*Lycoris longituba*, ^3^*Lycoris radiata*, ^4^*Narcissus papyraceus*, ^5^*Narcissus pseudonarcissus.* Abbreviations are given in the text.

Knowledge of the subcellular localization and organ-specific expression of genes and proteins involved in specialized metabolite biosynthesis provides information on its spatial organization and regulation (Watkins and Facchini, 2022). The overall compartmentalization and regulation of the alkaloid pathways have been well described in some medicinal plants such as *P*. *somniferum* or *C*. *roseus* (Watkins and Facchini, 2022), showing that there is no common compartmentalization and regulation to plants. This highlights the need for studies on compartmentalization in *Amaryllidaceae*. Enzyme subcellular localization and the gene expression pattern over different tissues and developmental stages have been described in *N*. *pseudonarcissus*, *L*. *radiata*, *L*. *longituba*, and *L*. *aestivum* (Fig. 2). *NBS* is expressed mainly in bulbs of *N*. *pseudonarcissus* sampled at the floral stage and in bulbs and roots of *L. aestivum* and *N*. *papyraceus* at the floral stage, while it is enriched in leaves of *L*. *longituba* sampled at the vegetative stage (Singh *et al.*, 2018; Li *et al.*, 2020; Tousignant *et al.*, 2022; Majhi *et al.*, 2023). The expression of *NR*, also involved in norbelladine synthesis, is higher in bulbs during the floral stage of *L. aestivum* and *N. papyraceus*, and during the vegetative stage of *L. radiata* (Park *et al.*, 2019; Majhi *et al.*, 2023), but is increased rather in the stem of *N*. *pseudornarcissus* during the floral stage (Singh and Desgagné-Penix, 2017). *N4OMT* was reported as expressed mainly in bulbs of *N*. *pseudonarcissus* and *L*. *radiata* in the vegetative stage, in bulbs and flowers of *N*. aff. *pseudonarcissus* at the floral stage, and in bulbs and roots of *L*. *longituba* in the vegetative stage (Kilgore *et al.*, 2014; Singh and Desgagné-Penix, 2017; Park *et al.*, 2019; Li *et al.*, 2020). In the case of *CYP96T1*, the highest expression was observed in the floral stems of *N*. *pseudonarcissus* in the floral stage, in roots and bulbs of *L*. *radiata* of the floral stage, and in bulbs of *L*. *longituba* in the vegetative stage (Singh and Desgagné-Penix, 2017; Park *et al.*, 2019; Li *et al.*, 2020). Overall, these studies suggest that these four key enzymes are often detected in bulbs, although there are differences between species and developmental stages. (Mehta *et al.*, 2024) performed a detailed tissue analysis and argued that biosynthesis of AA starts in the leaf bases (newly forming tissues in the bulb), where they detected expression of most of the genes responsible for AA biosynthesis, starting from 4'-*O*-methylnorbelladine (NBS and N4*O*MT were not included in that study). They propose that AA biosynthesis, starting from the phenol coupling reaction, primarily occurs in leaf bases. Even though more evidence is needed to prove that hypothesis, this conclusion is consistent with observations from alkaloid biosynthesis pathway in other plants, such as *Veratrum nigrum* and *Phlegmariurus tetrastichus* (Nett *et al.*, 2021; (Mehta *et al.*, 2024).

At the cellular level, *L. aestivum* and *N*. *papyraceus* NBS and NR are localized in the cytoplasm and nucleus (Majhi *et al.*, 2023), while *L*. *longituba* N4*O*MT is present only in the cytoplasm (Li *et al.*, 2020). CYP96Ts are membrane-bound proteins that are probably accumulating in the endoplasmic reticulum membrane, even though this has not been verified yet. Overall, these findings suggest that biosynthesis of AAs may start in the cytoplasm of cells of leaf bases. In the BIA pathway of *P*. *somniferum*, NCS and multiple other enzymes such as *O*MT were detected mainly in the phloem sieve elements, and NMTs in laticifers from leaves or stems; but gene expression principally happens in the phloem companion cells (Beaudoin and Facchini, 2014). Further studies need to be conducted to explore these aspects regarding AAs.

### Accumulation (storage) of alkaloids

Over 20 000 plants exude latex or mucilage upon physical damage or other interactions with the environment (Kekwick, 2002; Cui, 2019). The role of latex in storage and transport of alkaloids has been brought to light in medicinal plants such as *C. roseus* and *P. somniferum* (Beaudoin and Facchini, 2014; Watkins and Facchini, 2022). Plants of *Amaryllidaceae* secrete mucilage, which can cause skin irritations, when damaged by physical forces (Santucci and Picardo, 1992). In one study, narciclasine was isolated as a functional compound from the mucilage of *N*. *tazetta* which inhibited the seed germination and growth of rice and Chinese cabbage (Bi *et al.*, 1998). In addition to mucilage, vascular tissues, such as xylem and phloem, are involved in the transport and storage of precursors and alkaloids. Some major reactions of alkaloid biosynthesis have been detected inside these vascular elements (Watkins and Facchini, 2022). ‘Phloem sap’, or most probably mucilage, analysis of *Hippeastrum papilio* revealed that it was rich in galanthamine [30.2% of the total ion chromatogram (TIC)], haemanthamine (15.5% of TIC), and 11β-hydroxygalanthamine (3.6% of TIC) (Haist *et al.*, 2024).

Wang *et al.* (2007) studied the tissue distribution of galanthamine in *L*. *aurea* at the vegetative stage using fluorescent signals emitted by galanthamine They suggested that AAs may be stored in the apoplast of the tissues, mainly in the cell walls. According to the study, the primary organ of accumulation is leaf scales, and galanthamine is present in the cell walls of vascular bundles, mesophyll cells between vascular bundles, and epidermal cells of mature leaves. Multiple MS imaging (MSI) of the leaf cross-sections of *N*. *papyraceus* indicated that lycorine and 11-hydroxyvittatine are primarily found in the vicinity of vascular tissues, supporting the previous research on galanthamine accumulation (Mehta *et al.*, 2024). Furthermore, tissue staining with Dragendorff’s reagent of *H*. *papilio* indicated that alkaloids are more concentrated in vascular bundles, vacuoles, and intracellular spaces (Haist *et al.*, 2024). These studies indicate that AAs may be mainly produced in specialized cell types of vascular tissues, or in their proximity, and stored in the extracellular matrix such as the apoplast, highlighting the importance of the cellular transport of AAs.

### Transport (trafficking) of alkaloids

Takos and Rook (2013) suggested that AA glycosides, such as lycorine-1-*O*-β-d-glucoside, may be the form of transportation of AAs, increasing the solubility and minimizing the toxicity. Extracellular transport may be required to protect the plant from the toxicity of the produced specialized metabolites. Different transporters involved in alkaloid trafficking have been characterized in other medicinal plants such as *C*. *roseus*, and *Coptis japonica*. They fall under transporter families such as ATP-binding cassette (ABC), multidrug and toxic compound extrusion (MATE), and purine uptake permease (PUP) (Shitan *et al.*, 2014). There is only one published transporter related to *Amaryllidaceae* at present (R. Wang *et al.*, 2021). The ABC transporter ABCB11 is associated with the plasma membrane and transports lycorine outside of the cell in *L*. *aurea* (Fig. 2). An *in situ* hybridization technique revealed that this transporter is predominantly expressed in the phloem of leaves and bulbs, as well as in the cortical cells of roots of *L*. *aurea*, supporting the hypothesis that AAs are produced in cells of leaf bases and stored in the apoplast (R. Wang *et al.*, 2021). A comparative transcriptomic study related to methyl jasmonate (MJ) treatments, known to induce specialized metabolite production, showed changes in the expression level of 138 transporter genes. These transporters include ABC transporters (20; 14.49%), amino acid/peptide/protein transporters (23; 16.67%), and drug transporters (11; 7.97%). These changes could provide indications of AA transporters (Li *et al.*, 2021). In conclusion, further studies that combine the characterization of enzymes, transporters, and AAs *in planta* will provide a mechanistic understanding that will contribute to enhancing metabolic engineering possibilities.

## Insight into regulation of *Amaryllidaceae* alkaloid biosynthesis from *in vitro* culture studies

Field or greenhouse culture represents a simple approach for mass cultivation if not in competition with nutritional crops. It enables the control of environmental factors, such as nitrogen uptake, modulation of storage temperature, light wavelength, potting media, and application of fungicides, which may influence accumulation of specialized metabolites (El-Naggar and El-Nasharty, 2009; Lau *et al.*, 2014; Zaragoza-Puchol *et al.*, 2021). However, harvesting from cultivated *Amaryllidaceae* often leads to a lower yield compared with wild plants (Jin, 2013; Reis *et al.*, 2019) because our knowledge on regulation of the biosynthetic pathways is not complete. As an alternative to field- and greenhouse-grown plants, *in vitro* culture enables the exploration of the effect of many more factors simultaneously.

### Current methods of *in vitro* culture

*In vitro* culture was already used 70 years ago as a means of cell-free purified enzymes, from *Nerine bowdenii* flowering bulbs (Mann *et al.*, 1963). The following years were unsparing in different approaches and innovations. *In vitro* cultivation as a means for production of AAs is rather a long and contamination-prone process whose success depends on the species, the tissue and sample quality, the growth media, the time of acclimization, and many other unknown factors. The selection of the primary plant material (tissue and clone origin) appears to have a crucial effect on the AA yields (Bogdanova *et al.*, 2009; Georgiev *et al.*, 2020). This emphasizes the need for more efforts in the selection and study of high alkaloid-producing cultivars. It also suggests that AAs are produced by specialized differentiated cells of specific tissues, in response to environmental factors, and that modulation of their production is possible only within this frame.

Biotic and stresses have been the subject of numerous *in vitro* culture studies (Fig. 2). The application of fungal elicitors on *L. radiata* plant cultures induced the production of AA precursors (Zhou *et al.*, 2020). Bacterial synthetic communities applied to *in vitro* cultures of *L. radiata* suggested an interplay between AA production, bacterial endophytes, and fungal pathogens, and illustrated that AA biosynthesis could be better understood in the context of biotic interactions (Erb and Kliebenstein, 2020; Zhou *et al.*, 2023). Interestingly, a study reported that an *L. aestivum* endophytic bacterium *Paenibacillus lautus*, that was able to produce a wide range of plant hormones simultaneously, induced higher production of AAs by the plant but also endogenously produced its own, such as galanthamine, lycorine, ismine, lycoramine, galanthine, haemanthamine, homolycorine, 1,2-dihydrochlidanthine, and hippeastrine (Ptak *et al.*, 2022). Others have studied the effect of hormones such as jasmonic acid (JA) and 1-naphthaleneacetic acid (NAA), or specific light waves on different plant tissue (Fennell *et al.*, 2003; Kilgore and Kutchan, 2016; Berkov *et al.*, 2020; Ptak *et al.*, 2020; Meena *et al.*, 2022). Different auxins, picloram, meta-topolin, and thidiazuron were shown to regulate the regeneration rate and alkaloid profile in *L. aestivum*, *R. bifida*, and other species (Ptak *et al.*, 2017; Reis *et al.*, 2019). Specific combinations of hormones [6-benzylaminopurine (BAP), kinetin (KIN), and NAA] led to a specific AA increase in micropropagated *Caliphruria tenera* plants (Trujillo Chacón *et al.*, 2023). Treatment of an *in vitro* culture of *C. ×powellii* ‘Album’ with different conditions (light, dark, or auxins) led to variable tissue differentiation and growth, and a rather wide range of AAs such as lycorine, crinine, and cherylline types (Koirala *et al.*, 2023). In calli culture, light and auxin both modulated the production of many alkaloids, and AA biosynthetic genes *in vitro*, highlighting the delicate balance between stress and growth that must be achieved for calli to produce AAs.

All these studies emphasize the importance of discovering the biotic and abiotic elements that are involved in partial or complete activation of AA biosynthesis. Understanding the quality, quantity, and timing of the elicitors required to boost AA production is key to advance the yield range. In contrast to production for commercial purposes, the elucidation of the biosynthetic pathway does not require that AAs are produced in large amounts. It requires subtle differences in production between samples used in comparative omics studies. In this regard, harvesting samples from *in vitro* culture offers several advantages, such as homogeneous growth and controlled variables. The concomitant analysis of AA yield, biosynthetic genes, and culture conditions is the foundational knowledge that should be acquired to obtain a high yield of AAs in the future.

### Elucidation of biosynthetic pathways in *in vitro* culture

There are various environmental factors that can stimulate the production of alkaloids in plants. The previous section provided a non-exhaustive list of environmental stimuli used in *in vitro* culture that affect alkaloid biosynthesis in the *Amaryllidaceae* family. Transcription factors (TFs) are proteins that bind to specific DNA sequences, such as enhancer or promoter regions, initiating the transcription process that converts DNA to RNA. They coordinate the biosynthesis of specialized metabolites in response to environmental and developmental stimuli in plants (Ziegler and Facchini, 2008; Li *et al.*, 2024). There are many families of TFs studied in plants such as APETALA2/Ethylene-Responsive Factor (AP2/ERF), WRKY, and basic helix–loop–helix (bHLH) that contribute to alkaloid biosynthesis (Yamada and Sato, 2021). Also in upstream defence signalling, mechanisms such as JA signalling modulate the expression of TFs in response to environmental stresses (Wang *et al.*, 2023). Transcriptome analysis of various tissues of *L*. *longituba* revealed a high percentage of TFs such as bHLH, AP2/ERF, NAC, and TCP in this galanthamine-producing plant (Li *et al.*, 2020). A comparative transcriptomic study showed that MJ treatment was associated with an up-regulation of AA-related genes and of many TFs, such as WRKY (26 out of 32), AP2/EF (21 out of 25), and myeloblastosis (MYB) (all 14). As phenylpropanoid- and flavonoid-related genes were also up-regulated in this experiment, the identification of TFs specific to AA synthesis was not possible (Li *et al.*, 2021). Transcriptomic analysis related to floral development and anthocyanins in *L*. *chinensis*, *L*. *longituba*, *L*. *radiata*, and *L*. *sprengeri* identified multiple TFs, such as MYB, bHLH, AP2/ERF, Cys2–His2 zinc finger (C2H2), NAM, ATAF1/2, and CUC2 (NAC); however, this study did not focus on AA synthesis (Yue *et al.*, 2019; N. Wang *et al.*, 2021; F. Yang *et al.*, 2021; F. Zhang *et al.*, 2022). Transcriptomic analysis *N. pseudonarcissus* calli and field-grown plants also mentions the identification of different TFs (Ferdausi *et al.*, 2021). None of the identified sequences mentioned in all the literature detailed above are publicly available. Although most of these studies are related to anthocyanin or flavonoid synthesis in *Amaryllidaceae* plants, a deeper analysis could provide new insight into the AA pathway, as multiple specialized metabolite pathways are interconnected (Fig. 1). Recently, expression of heat shock factor (HSF) TFs was characterized in various tissues and flower developmental stages of *L. radiata*, and studied in response to hormones and abiotic stresses (Wang *et al.*, 2024). The expression of several *HSF* genes, especially *LrHSF5*, was associated with plant development and response to abiotic and hormone stresses. The correlation with AAs remains to be characterized further. Interestingly, a recent study on TFs related to MJ treatment in *L*. *aurea* helped identify a MYC TF (*La*MYC2) that up-regulated the biosynthesis of lycorine. The study demonstrated that *La*MYC2 binds to the E-box motifs of the promoter region of the *TYDC* gene of *L*. *aurea* involved in formation of the precursors of AA biosynthesis (Zhou *et al.*, 2024).

JA triggers the activation of TFs in response to environmental stresses (Goossens *et al.*, 2017). Jasmonate ZIM domain (JAZ) proteins are key components in the positive regulation of the interaction of JA signalling. In the *Amaryllidaceae* family, identification and characterization of JAZ genes have been performed in one study in *L*. *aurea* (Wang *et al.*, 2020). These authors cloned and characterized seven JAZ genes, and showed that the expression of the JAZ genes varied among tissues. Most of them were highly expressed in flowers, and JAZ 2, 5, and 6 were highly expressed in leaves. External MJ treatment up-regulated the expression of almost all of the JAZ genes and, at the protein level, JAZ 11 was expressed in both the nucleus and cytoplasm while JAZ 22 and 5 were expressed in the cytoplasm and JAZ 3, 4, 6, and 7 were expressed in the nucleus (Wang *et al.*, 2020). These authors have not studied the relationship of these JAZ genes with AA synthesis, but all the data (transcript, protein sequences) are available in public databases for further studies.

Until now, AA biosynthetic genes have been mostly elucidated one gene at a time at the molecular level, based on assumption of candidate genes identified by homology in transcriptomic data from a plant or its tissues grown in strictly specific conditions (Nguyen and Dang, 2021; Majhi *et al.*, 2023). This approach, although very useful, limits the discovery of the full potential of enzymes and of their physiological relevance. This is because the enzymes could be involved in multiple metabolic pathways and play important roles in their interaction.

*In vitro* culture offers a controlled platform that could help connect alkaloid, terpenoid, and phenolic compound pathways and reveal new ways to optimize AA production, but also understand the implication of AAs in cellular functions and defence-related mechanisms (Muro-Villanueva and Nett, 2023). Understanding the elements that modulate AA production would help identify specific conditions permissive or restrictive to their accumulation. These conditions and their transcriptomic and metabolomic consequences could be classified into biotic and abiotic elements, stored, and tracked in a database that would help researchers link triggers of AA production or of precursors, and thus understand new elements in the biosynthesis pathway.

## Available multi-omics information on *Amaryllidaceae* species

The genes involved in biosynthetic pathway may be organized in gene clusters, as is the case for several well-studied plant species, such as *Zea mays* (2,4-dihydroxy-l,4-benzoxazin-3-one and 2,4-dihydroxy-7-methoxy-l,4-benzoxazin-3-one biosynthesis; Frey *et al.*, 1997), *Oryza sativa* (momilactones and phytocassanes; Otomo *et al.*, 2004; Shimura *et al.*, 2007), *Papaver somniferum* (noscapine; Winzer *et al.*, 2012), *Arabidopsis thaliana* (thalianol and marneral; Field *et al.*, 2011), and *Solanum* spp. (terpenes; Matsuba *et al.*, 2013). Unfortunately, the resources that allowed the discovery and characterization of these gene clusters, such as linkage maps and genome assemblies, are lacking for *Amaryllidoideae* species.

### Genomic data

The cost of sequencing the nuclear genome of these species is prohibitive due to the large and complex genomes of members of this subfamily. For instance, their 1C genome size (which indicates the amount of DNA in a haploid nucleus) ranges from 6.03 Gbp in *Chlidanthus fragans* to 80.5 Gbp in *Galanthus lagodechianus* (Zonneveld *et al.*, 2003, 2005; Leitch *et al.*, 2019). In comparison, the *Z.mays* 1C genome is 2.65 Gbp and that of *A. thaliana* is 157 Mbp (Vu *et al.*, 2017; Leitch *et al.*, 2019). Also, their ploidy levels are so variable that the same species of the genus *Narcissus* has diploid, triploid, and tetraploid cultivars (Sochacki *et al.*, 2022), while the ploidy of the genus *Crinum* varies up to octoploid (Jones and Smith, 1967; Wahkstrøm and Laane, 2009). At present, the only genomes assembled and published in the *Amaryllidaceae* family are those of garlic (*Allium sativum*; Sun *et al.*, 2020) and onion (*Allium cepa*; Finkers *et al.*, 2021), both diploid species with genome sizes of 16.24 Gbp and 13.6 Gbp, respectively. However, the *Allioideae* subfamily does not produce AAs, limiting the interest in use of these recent genomic resources for the study of AA biosynthesis.

As regards *Amaryllidoideae*, a nuclear genome assembly for *Narcissus pseudonarcissus* was recently submitted to the NCBI Genome database (accession JAVXUK01). However, it may not be the final version, as it consists of 3 138 040 scaffolds, has no complete or partial chromosome, and no publication is associated with it. Also, four *Amaryllidaceae* genome sequencing datasets are available from the Ruili Botanical Garden (Liu *et al.*, 2019); however, the species from which the datasets originated was not provided. As these samples are part of the ‘10 000 Plant Genomes Project’ (Cheng *et al.*, 2018; https://db.cngb.org/10kp/), they should be clearly identified and the assemblies available in the near future. Finally, there are several chloroplast genome assemblies available for this subfamily, and they have been used for phylogenetic studies (Hori *et al.*, 2006; Jin *et al.*, 2018; Dennehy *et al.*, 2021; Konyves *et al.*, 2021; Zhang *et al.*, 2021; Cheng *et al.*, 2022).

### Transcriptomic and proteomic data

In the absence of genome assemblies, researchers have attempted to reconstruct the AA biosynthetic pathway using transcriptome sequencing combined with metabolomics or proteomics. Currently, there are transcriptomic data, in the form of raw reads or assembled transcriptomes, for 13 genera of this subfamily (Table 1), the first one being that of *Lycoris aurea* (Wang *et al.*, 2013). Unfortunately, these data are not always made publicly available (Chang *et al.*, 2011; Wang *et al.*, 2017; Song *et al.*, 2019; Xiang *et al.*, 2022). In other cases, accession numbers or links to their datasets/assemblies are not provided (Pulman, 2014; Ferdausi *et al.*, 2021) or contain mistakes (J. Yang *et al.*, 2021, 2023; Ren *et al.*, 2022), complicating the analysis. Park *et al.* (2019) published their transcriptome assembly for *L. radiata* in NCBI SRA, without the raw data, while J. Yang *et al.* (2021) reported the use of long- and short-read technology for the generation of a high-quality transcriptome assembly of *Narcissus tazetta*, but neither the final assembly nor the raw long reads were provided. As the use of long-read sequencing is new for *Amaryllidoideae* species, these datasets and assemblies should be published since they could help transcriptome and, in the future, genome annotations.

**Table 1.** Transcriptomic studies from *Amaryllidoideae* species

Species	Reference	Raw data	Assembly	Tissue	Developmental stage	Metabolomics
*Amaryllis belladonna*	One Thousand Plant Transcriptomes Initiative (2019)	ERR2040723	LDME^a^	Stem or leaf	NA	NA
*Clivia miniata*	Q.M. Wang *et al.* (2018)	PRJNA480383	NA	Leaf	Mature striped plants, young leaves	NA
	Y. Li *et al.* (2022)	PRJNA813401	NA	Fruit peel, flower, leaf	NA	Anthocyanins, flavonoids, terpenes
*Crinum*×*powellii*	Koirala *et al.* (2023)	PRJNA962562	Koirala *et al.* (2023)	Leaf, bulb, basal plate, root, callus	Undifferentiated callus and 4-week-old plants	AA precursors and AAs
*Galanthus elwesii*	Kilgore *et al.* (2016a)	PRJNA306697	^b^	Leaf, bulb, inflorescence	Adult, blooming	Galanthamine
*G. sp. MBK-2015*	Kilgore *et al.* (2016a)	PRJNA306273	^b^	Leaf, bulb, inflorescence	Adult, blooming	Galanthamine
*Hippeastrum hybrid cultivar*	X. Li *et al.* (2022)	PRJNA608969	NA	Stamen	3-year-old plants, blooming	NA
	Y. Wang *et al.* (2018)	PRJNA322243	NA	Flower	NA	NA
*H. vittatum*	–	PRJNA862291	NA	Bud	–	
*Leucojum aestivum*	Tousignant *et al.* (2022)	PRJNA720900	NA	Bulb	Dormant	AAs
*Lycoris aurea*	Wang *et al.* (2013)	PRJNA188333	NA	Stem, flower, leaf	Bud, blooming, wilting	NA
	Ren *et al.* (2022)	PRJNA574869^c^ PRJNA579847^c^	NA	Bulb	Cross-cut bulb to bulblet formation	Untargeted metabolomics, sugar content, JA, ABA, and ethylene
*L. chinensis*	F. Zhang *et al.* (2022)	PRJNA847051	NA	Shoot apical meristem	1- to 4-year-old bulbs	NA
*L. incarnata*	–	PRJNA639315	NA	Bulb	Vegetative stage	NA
*L. longituba*	F. Zhang *et al.* (2022)	PRJNA490415	NA	Tepal	Small, medium, and opening bud	Floral volatile organic compounds, anthocyanins
	Li *et al.* (2020)	PRJNA590043	NA	Bud, leaf, root	3 years old	Galanthamine
	Li *et al.* (2021)	PRJNA720237^c^	NA	Seedling	7 d old	AA precursors and AAs
*L. radiata*	N. Wang *et al.* (2021)	PRJCA006232^d^	NA	Petal	Flowering	Protoanthocyanidins and anthocyanins
	Park *et al.* (2019)	NA	PRJNA529664	Bud, leaf, root	NA	Primary metabolites and galanthamine
	Xu *et al.* (2020)	PRJNA574731	NA	Bulb	Dormancy, competence, bud initiation, bud enlargement, bulblet emergence and bulblet development	Hormones, starch, and soluble sugar
*L. sprengeri*	F. Yang *et al.* (2021)	PRJNA714286	NA	Petal	Adult, blooming	Anthocyanins, flavonoid-biosynthesis related metabolites, and brassinolide
	Ren *et al.* (2022)	PRJNA574869^c^ PRJNA579847^c^	NA	Bulb	Cross-cut bulb to bulblet formation	Untargeted metabolomics, sugar content, JA, ABA, and ethylene
*Narcissus papyraceus*	Hotchandani *et al.* (2019)	PRJNA407433	NA	Bulb	Dormant	Heterocyclic compounds, lycorine
*N. pseudonarcissus*	Ferdausi *et al.* (2021); Pulman (2014)	PRJNA264603	NA	Basal plate and callus	4 months after planting	NA
	Singh and Desgagné-Penix (2017)	PRJNA392294	NA	Bulb	Adult, blooming	AAs, galanthamine
	X. Li *et al.* (2018)	PRJNA497707	NA	Perianth and corona	S4 flowering stage	Carotenoids
*N. aff. pseudonarcissus*	Kilgore *et al.* (2014)	PRJNA301357	^b^	Leaf, bulb, inflorescence	Adult, blooming	Galanthamine
*N. tazetta*	Yang *et al.* (2023)	PRJNA891931^c^	NA	NA	NA	Flavonoids, proanthocyanins, and anthocyanin
	Y. Zhang *et al.* (2022)	PRJNA855612	NA	Corolla and tepal	From bud to decay	Carotenoid and flavonoid content
	–	PRJNA296436	NA	Yellow petal and yellow corona	Early flowering	
	Ren *et al.* (2017)	PRJNA340092 PRJNA340090	NA	Tepal	5, 12, and 20 d after planting	Chlorophyll, carotenoids, and flavonoids
	G. Wang *et al.* (2018)	PRJNA387061	NA	Basal plate	3-year-old bulbs	Flavonols
	Yan-Hong *et al.* (2019)	PRJNA487120	NA	Bulb	3-year-old bulbs	NA
	He *et al.* (2020a, b)	PRJNA523125	GSE126727	Corolla and petal	Early flowering and full bloom	NA
	J. Yang *et al.* (2021)	SUB10083597^c^ PRJNA750844	NA	Corona and tepal	Adult, blooming	Flavonoids, carotenoids, chlorophyll, and volatile organic components
*N. viridiflorus*	One Thousand Plant Transcriptomes Initiative (2019)	ERR2040725 ERR2040724	IQYY, XEUV, TRRQ^a^	Young vegetative tissue and flower	NA	NA
*Phycella aff. cyrtanthoides*	One Thousand Plant Transcriptomes Initiative (2019)	ERR3487375	DMIN^a^	Young vegetative tissue	NA	NA
*Rhodophiala pratensis*	One Thousand Plant Transcriptomes Initiative (2019)	ERR2040726	JDTY^a^	Young vegetative tissue	NA	NA
*Traubia modesta*	One Thousand Plant Transcriptomes Initiative (2019)	ERR2040727	ZKPF^a^	Young vegetative tissue	NA	NA
*Zephyranthes candida*	Y. Zhang *et al.* (2022)	PRJNA796382	NA	Flower and flower stalk	Adult, blooming	NA
*Z. treatiae*	One Thousand Plant Transcriptomes Initiative (2019)	ERR2040728	DPFW^a^	Young vegetative tissue	NA	NA

The accession number of the raw data, the assembly accession, the tissue and developmental stage sampled, and the metabolites quantified are included.

AA, *Amaryllidaceae* alkaloid; NA, not available

^*a*^ Assembly available at GigaScience Database (http://gigadb.org/dataset/100627).

^*b*^ Assembly available in the MedPlant RNASeq Database (https://medplantrnaseq.org).

^*c*^ The wrong acces sion number was provided.

^*d*^ Data available on https://ngdc.cncb.ac.cn/.

All transcriptome studies presented here were done using bulk RNA-seq. In some cases, a single tissue was sampled (Singh and Desgagné-Penix, 2017; Y. Wang *et al.*, 2018; Hotchandani *et al.*, 2019; One Thousand Plant Transcriptomes Initiative, 2019; Tousignant *et al.*, 2022; Xiang *et al.*, 2022). In others, tissue samples were pooled into a single library (Y. Li *et al.*, 2022; Wang *et al.*, 2013; C.H. Zhang *et al.*, 2022). These tactics allowed the identification of several genes in the AA biosynthetic pathway, as well as genes in anthocyanin and phenylpropanoid pathways, and were sufficient for phylogenetic studies (Y. Wang *et al.*, 2018; One Thousand Plant Transcriptomes Initiative, 2019). However, genes weakly expressed in a single tissue or cell type may have been missed. Coupling the study of multiple tissues and conditions with metabolomics enables co-expression analyses, using known genes of the pathway as bait to pull out new candidates from the transcriptome (Kilgore *et al.*, 2014, 2016a; Koirala *et al.*, 2023). This is potentiated by single-cell multi-omics, which was recently used in *C. roseus* and led to the identification of a new enzyme in the monoterpene indole alkaloid pathway (Li *et al.*, 2023).

Comparative proteomic studies can help identify candidate enzymes in the biosynthetic pathway by analysing species that differ in their alkaloid composition. Of the three proteomic studies available for *Amaryllidoideae*, all of them have analysed *Lycoris* species (Ru *et al.*, 2013; Jiang *et al.*, 2021; Tang *et al.*, 2023). The study by Tang *et al.* (2023) was the only one that focused on alkaloid biosynthesis. By comparing *L*. *longituba*, *L*. *sprengeri*, and *L*. *incarnata*, the authors were able to identify candidates for N4*O*MT and for the *N*-methyltransferase that converts norgalanthamine into galanthamine, but the sequences of these enzymes have not been published.

### Multi-omics

In upcoming omics research, integrating transcriptomics and proteomics to compare tissues or populations with varying alkaloid contents (as reported for *G. elwesii*; Berkov *et al.*, 2004) will be essential. It will determine whether the differences in alkaloid content are predominantly influenced by variations in the genes expressed, their expression levels within specific tissues or populations, or if these metabolic distinctions can be attributed to translational or post-translational mechanisms. Once genome sequencing becomes a more affordable avenue in the study of *Amaryllidoideae* species, transcriptomic and proteomic studies will help improve genome annotations. Omics toolsets also offer a powerful approach to study genetic polymorphism, evolution, and single nucleotide polymorphism in homologous genes between *Amaryllidaceae* species, and their link to present/absent enzymatic reactions and related metabolites (Stander *et al.*, 2022; Méteignier *et al.*, 2023). Then, comparative studies between species with different alkaloid compositions, or accumulating specific alkaloids in different amounts, will facilitate the search for the missing enzymes of the AA biosynthesis pathway. Furthermore, comparative genomics and phylogenetic analysis will help elucidate the evolutionary relationships between alkaloid biosynthetic pathways in different plant families, aiding in predicting undiscovered enzymes and pathways.

## Importance of prediction tools and databases

### Prediction tools of biosynthetic pathways and metabolic routes

Prediction tools, such as Plant Metabolic Network 15, RefMetaPlant, and MetaCyc, that forecast metabolic routes are improving the discovery of new pathways in many aspects (Caspi *et al.*, 2020; Hawkins *et al.*, 2021; Shi *et al.*, 2024). The PathPred on Genome Japan tools helps predict pathways by machine learning (Moriya *et al.*, 2010). Prediction deep learning tools could also help to discover uncharacterized plant metabolites. For example, searching for potent therapeutical compounds with similarities to AAs could help to suggest undiscovered AAs and identify their value (Sreeraman *et al.*, 2023). A recent article describes a Self-driving Autonomous Machines for Protein Landscape Exploration (SAMPLE) platform designed to combine prediction and experimental automation to engineer proteins with zero human intervention for synthetic biology and pathway elucidation (Rapp *et al.*, 2024). These new platforms provide insight for future scientific discoveries.

### Public databases for *Amaryllidaceae* alkaloids

Many facets of AA biosynthesis are being covered in public database, including a TF database PlantTFDB 4.0 (Jin *et al.*, 2016), Gene Ontology annotation through Planteome (Cooper *et al.*, 2024), metabolomes through PMhub 1.0 or RefMetaPlant (Shi *et al.*, 2024; Tian *et al.*, 2024), transport through ChannelsDB (Špačková *et al.*, 2024), and many more. However useful, these databases are not sufficient on their own to decipher AA biosynthetic pathways. A collaborative effort involving multiple research studies has been integrated into a single database that includes genomes of a few reference species, transcriptomic data in different conditions, and—most importantly—AA profiling in all available experiments. To understand the physiological fate of AAs and improve metabolic engineering strategies, data from proteomic analysis, *in vitro* assays, and propagation yield results according to various conditions should be further included. Such a united effort would allow gathering and visualizing valuable datasets in one platform, similarly to TAIR for Arabidopsis, The Bio-Analytic Resource for Plant Biology BAR, and Genevestigator for multiple species (Hruz *et al.*, 2008; Lamesch *et al.*, 2012; Waese and Provart, 2017). Building a network of AA researchers would not only allow cost sharing but also build stronger datasets and exchange of expertise and resources. Furthermore, multi-omic metadata gathered in a single platform along with datasets on biotic and abiotic experiments, phenotyping, and chemical profiling would allow faster discovery of AA enzymes and improve our definition of *Amaryllidaceae* plant interactions with their environment, a very useful piece of the puzzle to add to biosynthetic pathway discovery, and to *in vitro* production for higher AA production.

## Conclusion

Knowledge of the complex genetic regulation, transport, and accumulation of AAs would solve complex questions concerning the chronological order in their synthesis and allow further technological advances such as metabolic engineering of *in vitro* tissues or heterologous systems.

In conclusion, the production of *Amaryllidaceae* alkaloids is a fascinating and intricate process that offers numerous opportunities for exploration and discovery. Studying these pathways not only sheds light on plant biochemistry, but also has implications for pharmacology and potential medicinal uses of these alkaloids. There is still much to uncover regarding the specialized metabolite production in *Amaryllidaceae* plants, such as identifying new enzymes, improving their activity, and understanding the interconnected pathways. The ongoing research in this area holds the possibility to unleash the full potential of these bioactive compounds for medicinal, agricultural, and industrial purposes.

## Abbreviations:

AA: *Amaryllidaceae* alkaloid
BIA: benzyl isoquinoline alkaloid
C*O*MT: catechol-*O*-methyltransferase
3,4-DHBA: 3,4-dihydroxybenzaldehyde
NBS: norbelladine synthase
NCS: norcloclaurine synthase
N*O*MT: norbelladine-*O*-methyltransferase
N4OMT: norbelladine 4'-*O*-methyltransferase
NR: noroxomaritidine reductase
SDR: short-chain dehydrogenase
